# Supplemental Transmission Aided Attenuation Correction for Quantitative Cardiac PET

**DOI:** 10.1109/TMI.2023.3330668

**Published:** 2024-03-05

**Authors:** Mi-Ae Park, Vlad G. Zaha, Ramsey D. Badawi, Spencer L. Bowen

**Affiliations:** Department of Radiology, UT Southwestern Medical Center, Dallas, TX 75390 USA; Cardiology Division, Department of Internal Medicine, the Advanced Imaging Research Center, and the Harold C. Simmons Comprehensive Cancer Center, UT Southwestern Medical Center, Dallas, TX 75390 USA; Department of Radiology, University of California Davis Medical Center, Sacramento, CA 95817 USA; Department of Radiology, UT Southwestern Medical Center, Dallas, TX 75390 USA

**Keywords:** Attenuation correction, cardiac, positron emission tomography-magnetic resonance (PET/MR), positron emission tomography-computed tomography (PET/CT), transmission acquisition

## Abstract

Quantitative PET attenuation correction (AC) for cardiac PET/CT and PET/MR is a challenging problem. We propose and evaluate an AC approach that uses coincidences from a relatively weak and physically fixed sparse external source, in combination with that from the patient, to reconstruct μ-maps based on physics principles alone. The low 30 cm^3^ volume of the source makes it easy to fill and place, and the method does not use prior image data or attenuation map assumptions. Our supplemental transmission aided maximum likelihood reconstruction of attenuation and activity (sTX-MLAA) algorithm contains an attenuation map update that maximizes the likelihood of terms representing coincidences originating from tracer in the patient and a weighted expression of counts segmented from the external source alone. Both external source and patient scatter and randoms are fully corrected. We evaluated performance of sTX-MLAA compared to reference standard CT-based AC with FDG PET/CT phantom studies; including modeling a patient with myocardial inflammation. Through an ROI analysis we measured ≤5% bias in activity concentrations for PET images generated with sTX-MLAA and a TX source strength ≥12.7 MBq, relative to CT-AC. PET background variability (from noise and sparse sampling) was substantially reduced with sTX-MLAA compared to using counts segmented from the transmission source alone for AC. Results suggest that sTX-MLAA will enable quantitative PET during cardiac PET/CT and PET/MR of human patients.

## I. Introduction

PET/CT is an important tool in the clinical workup of patients with cardiac diseases. Separately, combined PET/MR has shown advantages over conventional imaging for several cardiac applications (e.g. diagnosis of myocardial inflammation). Quantitative PET metrics provide benefits in both cases. For PET/CT, absolute measurements of myocardial flow reserve have superior diagnostic sensitivity to semi-quantitative analysis alone for detecting coronary artery disease. On PET/MR, quantification of uptake aids in the evaluation of myocardial inflammation [1], [2], [3].

Correcting for patient-related factors is necessary to minimize artifacts and produce quantitative PET images during cardiac exams. Failure to correct for 511 keV photon attenuation typically has the largest impact, of all data corrections, on PET quantification. However, for PET/CT the CT used for attenuation correction (AC) has limited diagnostic benefit; contributing 0.5–1 mSv of effective dose (compared with 2 mSv from ^13^N-ammonia alone) [4]. With PET/MR, MR-based methods (MR-AC) have significant performance and/or practical limitations in cardiothoracic imaging. For instance, 50% of patients had μ-maps with MR susceptibility artifacts in one study [5], frequently due to cardiac stents (resulting in differences of up to 200% in SUVs relative to PET reconstructions with manually corrected μ-maps). Deep learning algorithms have benefited MR-AC [6] and represent the current state of the art approaches [7]. But, bias in PET images was frequently >10% for a generative adversarial network scheme directly converting MR images to μ-maps [8]. For both modalities, respiratory mismatch between the MR or CT (typically breath hold) and PET (shallow breathing) exams, can result in PET artifacts throughout the thoracic cavity [5].

As an alternative to CT-AC and MR-AC, methods have been developed that utilize the PET signal to reconstruct μ-maps. A common algorithm, using counts from the patient alone, is termed maximum likelihood reconstruction of activity and attenuation (MLAA). However, MLAA methods suffer cross-talk between emission (EM) image and μ-map estimates, only reconstruct attenuation values up to a constant (requiring a prior to correct for [9] and [10]), and frequently rely on CT images for scatter correction or normalization compensation (not practical on PET/MR); resulting in PET image bias over 10% relative to CT-AC [11]. To address these limitations, researchers have used the intrinsic ^176^Lu radiation from PET detectors (i.e. alone or to produce an initial μ-map for MLAA) [12] or events from a hollow cylinder-shaped source alone [13]. However, the relatively low count rates from ^176^Lu [12], [14], [15] or the external source, combined with the challenges in correcting for the scatter of ^176^Lu gamma rays [12] resulted in PET bias exceeding 5% in thoracic ROIs. Furthermore, filling and placing a hollow cylinder-shaped source that occupies the full PET field-of-view (FOV) is challenging. Deep learning has been used to aid MLAA [16], estimate μ-maps from EM data, or convert uncorrected EM images to AC results [17]. However, tracer bias also frequently exceeds 5% with these methods.

Panin et al. [18] developed an algorithm that uses counts from a rotating external positron source, combined with those from the tracer in the patient, to correct for the deficits of MLAA. The rationale is that the higher count rate, for coincidences from the patient, combined with the more accurate attenuation estimation using coincidences from the external source, can produce AC PET images with higher signalto-noise ratio (SNR) and accuracy, compared to using either strategy alone. The method of Panin et al, however, utilized PET data from 10 hr exams to correct for scatter from the external source (not practical for patient PET/MR exams). Furthermore, tight space confines of whole body PET/MR scanners prevent integration of a rotating transmission source. We propose a supplemental transmission (TX) aided MLAA (sTX-MLAA) approach that uses 1) a physically fixed source, placed near the bore, that can be relatively easily filled and placed, 2) a transmission update that combines signal from the patient and external source, and 3) data corrections for TX source and patient scatter, to estimate and apply AC using PET data acquired during the exam intended for correction alone. The purpose of this study is to develop and evaluate sTX-MLAA with anthropomorphic phantom experiments approximating cardiac PET exams.

## II. Methods

### A. Overview

Phantom acquisitions were performed on a time-of-flight (TOF) capable whole-body PET/CT; Siemens Biograph mCT Flow PET/CT (Siemens Medical Solutions USA, Inc). We used a PET/CT to facilitate a direct comparison with the reference standard: PET images corrected for attenuation with CT. Our approach uses a custom TX source, detailed in Section II–B. We then describe the supplemental transmission aided attenuation map reconstruction algorithm, including its theory (Section II–C.1), PET data pre-processing (Section II–C.2), method for updating and estimating the emission images (Section II–C.3), approach for attenuation map updates (Section II–C.4), the combined update equation (Section II–C.5), and data corrections (Section II–C.6). Pseudo-code for the complete sTX-MLAA scheme, including scatter corrections, is shown in Algorithm 2. Phantom experiments, data processing, and analysis are detailed in Section II–D. ^18^F-fluorodeoxyglucose (FDG) was used for filling the TX source in all experiments.

### B. Sparse Transmission Source

**Fig. 1.**
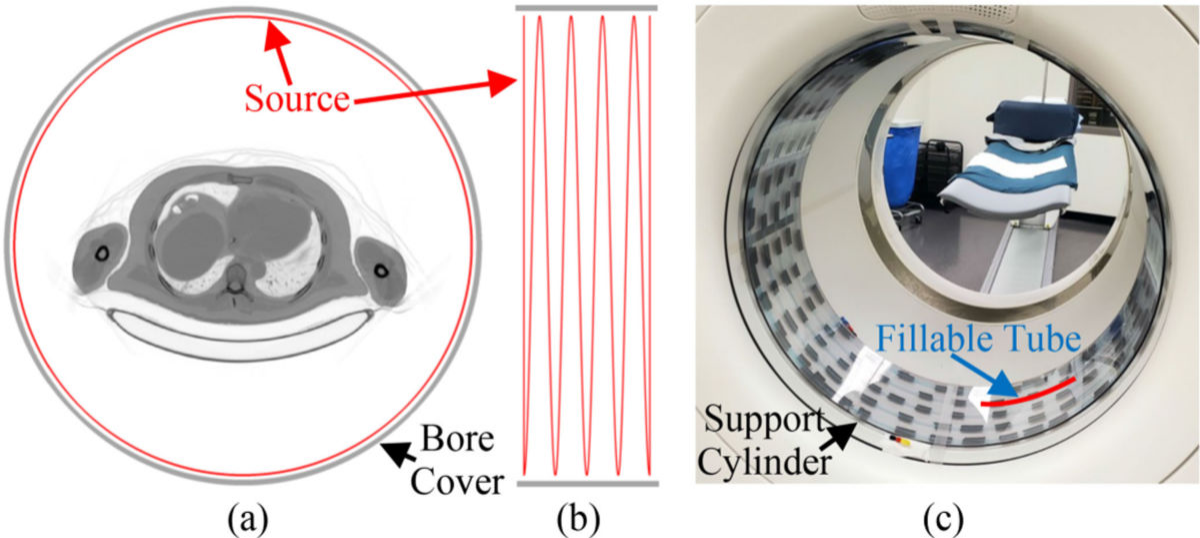
Overview of the sparse transmission source. Schematic of the tori-helix geometry in the (a) transverse and (b) sagittal planes, fused with a CT image from a human patient for reference. (c) The source prototype placed in the bore of the PET/CT used for this study.

A sparse transmission source was constructed by wrapping a polytetrafluoroethylene (PTFE) fillable tube (ID=1.6 mm) around the outside of a hollow cylinder-shaped support (OD=76.2 cm, ID=75.2 cm, height=25.4 cm), made of polycarbonate. Polycarbonate annuli (ID=76.2 cm, OD=78 cm) were fitted on either end of the support cylinder to center the TX source in the transverse FOV. Fig. 1 shows the prototype setup. We note that this configuration only reduces the ID of the Biograph mCT bore cover by 2.8 cm, which creates minimal mechanical interference when imaging patients that occupy a large fraction of the FOV. Additionally, by placing the source near the bore cover, the interference between coincidences originating from the TX source and patient is minimized.

The geometry of the fillable tube was chosen as a tradeoff between tomographic sampling, TX source count rate, impact on tracer reconstruction SNR, and ease of filling. Specifically, simulations demonstrated that sparse geometries can produce a higher noise equivalent count rate (surrogate for image SNR^2^) compared to rotating rods or a uniformly filled hollow cylinder-shaped source, at equal TX singles flux [19], [20]. Thus, we utilized two tori, near the axial ends of the PET FOV, joined with a four turn helix (pitch=5.3 cm), over a total axial length of 22 cm (PET axial FOV=22.1 cm). End-plane tori assist in reducing limited sampling artifacts that occur when using a helix alone. The PTFE tube had a total length of ~14.4 m, fill volume of 30 cm^3^, and was fitted with Luer adapters (syringe connections) on the ends for easy filling and draining.

### C. Supplemental Transmission Aided Attenuation Map Reconstruction

#### 1) Theory:

The expected prompts ${\bar{y}}_{it}(\mu ,x)$, as a function of the attenuation map (μ) and patient radiotracer (x) image estimates, when scanning with a TX source, is given by

(1) $${\bar{y}}_{it}(\mu ,x)=\left[b_{it}+\frac{\sum_{j}P_{itj}x_{j}}{N_{i}}\right]a_{i}+\frac{\left({\bar{s}}_{it}(b)+{\bar{s}}_{it}(x)\right)}{N_{i}}+{\bar{r}}_{i}$$

(2) $$a_{i}=\exp\left(l_{i}\right)=\exp\left(-\sum_{j}A_{ij}\mu_{j}\right)$$

where $b_{it}$ is the measured blank projection counts from the TX source (acquired without the patient in the PET scanner) for 3D sinogram coordinate i and TOF index t, j is the image voxel index, $P_{itj}$ are the elements of the TOF projection matrix, $N_{i}$ are the normalization sinogram factors, $a_{i}$ is the set of attenuation factors as a function of 3D sinogram coordinate alone, ${\bar{s}}_{it}(b)$ is a sinogram of expected scattered counts from the TX source only, ${\bar{s}}_{it}(x)$ represents expected scatter attributed to tracer in the patient, ${\bar{r}}_{i}$ is a sinogram of the random coincidence estimates (constant for all TOF indices at a given line-of-response *i*), $A_{ij}=\sum_{t}P_{itj}$ are elements of the non-TOF projection matrix, and $l_{i}$ are the product of projections lengths and linear attenuation coefficients (LACs). We note that the emission image, x, accounts only for radiotracer in the patient alone (i.e. excluding the external source). It is assumed that the blank projection $\left(b_{it}\right)$ has been corrected for radionuclide decay, dead-time, and scan duration to match the scan with the patient.

Separately, by applying sinogram radial thresholding (implemented in Section II–C.2), we can estimate prompts originating from the TX source alone $({\bar{y}}_{i}^{{\text{TX}}_{\text{sep}}})$, as originally proposed by Mollet et al. [13]. Specifically, using the mask, $m_{it}^{{\text{TX}}_{\text{sep}}}$, we sum TOF bins at sinogram projection elements that spatially intersect the external TX source. Expected counts are described as follows:

(3) $${\bar{y}}_{i}^{{\text{TX}}_{\text{sep}}}(\mu )=b_{i}^{{\text{TX}}_{\text{sep}}}a_{i}+\frac{{\bar{s}}_{i}\left(b^{{\text{TX}}_{\text{sep}}}\right)}{N_{i}}+{\bar{r}}_{i}^{{\text{TX}}_{\text{sep}}}$$

where $b_{i}^{{\text{TX}}_{\text{sep}}}$ is the blank sinogram processed with the radial thresholding operation $\left(b_{i}^{{\text{TX}}_{\text{sep}}}=\sum_{t}m_{it}^{{\text{TX}}_{\text{sep}}}b_{it}\right)$, ${\bar{s}}_{i}\left(b^{{\text{TX}}_{\text{sep}}}\right)$ are elements of the estimated scatter for segmented TX counts alone, and ${\bar{r}}_{i}^{{\text{TX}}_{\text{sep}}}$ are expected randoms for the radially thresholded coincidences. The overall assumption is that net true coincidences (sum of trues and scatters) in ${\bar{y}}_{i}^{{\text{TX}}_{\text{sep}}}$ are only counts from the TX source impacted by the μ-map, and there is negligible interference from patient annihilation photons.

To estimate the μ-map and EM image (x) we use the log-likelihood of the PET data. The equation combines 1) measured prompts from the patient and the TX source $\left(y_{it}\right)$ and 2) the radially thresholded counts from the TX source alone $\left(y_{i}^{{\text{TX}}_{\text{sep}}}\right)$. Assuming the PET data represent independent Poisson random variables, the complete log-likelihood equation is given by

(4) $$L(y|\mu ,x)=\left[\sum_{i}\sum_{t}y_{it}\log\left({\bar{y}}_{it}(\mu ,x)\right)-{\bar{y}}_{it}(\mu ,x)\right]+\alpha \left[\sum_{i}y_{i}^{{\text{TX}}_{\text{sep}}}\log\left({\bar{y}}_{i}^{{\text{TX}}_{\text{sep}}}(\mu )\right)-{\bar{y}}_{i}^{{\text{TX}}_{\text{sep}}}(\mu )\right]-\beta R(\mu )$$

where α is a hyperparameter that adjusts the strength of the term in (4) that represents the likelihood of radially thresholded counts from the TX source, β is a hyperparameter, and R(μ) is a roughness penalty function. The α hyperparameter balances the impact of counts originating from the patient and those from the TX source; compensating for the sparse tomographic sampling and typically lower count rates, relative to the patient, of the TX source. Panin et al. [18] optimized (4) with α=0, but as they found and as we show here, this is often inadequate for accurate AC with a physically fixed and sparse TX source. Cheng et al. [21] developed an MLAA algorithm by optimizing a similar expression to (4), largely focused on using the gamma rays from ^176^Lu in the term weighted by α. However, their method did not include scatter estimations (i.e. was assumed known), did not account for key methodological aspects of using an external TX source during patient scans (e.g. pre-processing and in image updates), and was almost entirely evaluated on simulations. This study innovates by developing the sparse source, joint reconstruction, and scatter corrections, specifically for TX source-aided AC. Further, it includes a comprehensive experimental evaluation on physical phantoms.

To estimate the EM image (x) and μ-map (μ) maximizing the likelihood in (4), we developed an iterative algorithm that alternatively updates EM and μ-map values, including scatter correction. We detail these methods in the following sections.

#### 2) Data Pre-Processing:

To estimate coincidences originating from the TX source and patient alone we employed a radial thresholding mask $\left(m_{it}^{\text{Radial}}\right)$ in sinogram space, described as follows:

(5) $$\begin{array}{l} m_{it}^{\text{Radial}}\left(d^{\text{L}},d^{\text{U}}\right)\\ =\{\begin{array}{l} 1,\;d^{\text{L}}\leq d(i,t)\leq d^{\text{U}}\\ 0,\;\text{otherwise} \end{array} \end{array}$$

(6) $$\begin{array}{l} d(i,t)\\ =\sqrt{d^{\text{D}}\sin{\left(\left[i\text{\%}n_{p}-p_{0}\right]\text{Δ}p/d^{\text{D}}\right)}^{2}+{\left[t-t_{0}\text{Δ}t\right]}^{2}} \end{array}$$

where $d^{\text{L}}$ and $d^{\text{U}}$ are the lower and upper radial limits, respectively, $d^{\text{D}}$ is the average distance from the center FOV to the detector, % is the modulo operator, $n_{p}$ is the number of radial projection bins, Δp is the projection bin size, $p_{0}$ and $t_{0}$ are central bins, and Δt the TOF sampling in space. Masks to segment coincidences originating from the TX source $\left(m_{it}^{{\text{TX}}_{\text{sep}}}\right)$ or patient alone $\left(m_{it}^{{\text{EM}}_{\text{sep}}}\right)$ were produced as follows:

(7) $$m_{it}^{{\text{TX}}_{\text{sep}}}=m_{i}^{\text{Triple}}m_{it}^{\text{Radial}}(340\;\text{mm},480\;\text{mm})$$

(8) $$m_{it}^{{\text{EM}}_{\text{sep}}}=m_{it}^{\text{Radial}}(0\;\text{mm},340\;\text{mm})$$

where $m_{i}^{\text{Triple}}$ is a mask used to employ the “triple-point” method; reducing the impact of scattered and randoms coincidences from the TX source. The mask selects lines-of-response (LORs) that intersect the TX source, and was produced by intensity thresholding the blank sinogram. Radial limits were chosen largely to minimize interference of counts from the patient on those from the TX source (at a radius of 38.3 cm), as these were often much higher in magnitude. Segmented coincidences were produced with the masks as follows:

(9) $$y_{it}^{{\text{EM}}_{\text{sep}}}=m_{it}^{{\text{EM}}_{\text{sep}}}y_{it},b_{it}^{{\text{EM}}_{\text{sep}}}=m_{it}^{{\text{EM}}_{\text{sep}}}b_{it}$$

(10) $$\begin{array}{l} y_{i}^{{\text{TX}}_{\text{sep}}}=\sum_{t}m_{it}^{{\text{TX}}_{\text{sep}}}y_{it},b_{i}^{{\text{TX}}_{\text{sep}}}=\sum_{t}m_{it}^{{\text{TX}}_{\text{sep}}}b_{it},\\ {\bar{r}}_{i}^{{\text{TX}}_{\text{sep}}}={\bar{r}}_{i}\sum_{t}m_{it}^{{\text{TX}}_{\text{sep}}} \end{array}$$

where $y_{it}^{{\text{EM}}_{\text{sep}}}$ and $b_{it}^{{\text{EM}}_{\text{sep}}}$ are measured prompt and blank counts, respectively, segmented around the patient, and ${\text{TX}}_{\text{sep}}$ superscript sinograms are segmented around the TX source.

### 3) Emission Reconstruction:

For EM image reconstructions and updates we used TOF-based Ordinary Poisson ordered subsets expectation maximization (OP-OSEM). Subset updates are given by

(11) $${\hat{x}}_{j}^{n,m+1}=\frac{{\hat{x}}_{j}^{n,m}}{\sum_{i\in S_{m},t}\frac{P_{itj}}{N_{i}}}\sum_{i\in S_{m},t}P_{itj}\phi_{itj}^{n,m}$$

(12) $$\phi_{itj}^{n,m}=\{\begin{array}{ll} {\text{Δ}}_{itj}^{n,m}, & \text{if}\frac{P_{itj}{\hat{x}}_{j}^{n,m}}{b_{it}^{{\text{EM}}_{\text{sep}}}}>0.1\\ 0, & \text{otherwise} \end{array}$$

(13) $${\text{Δ}}_{itj}^{n,m}=\frac{y_{it}^{{\text{EM}}_{\text{sep}}}}{a_{i}\left(\sum_{j}P_{itj}{\hat{x}}_{j}^{n,m}+N_{i}b_{it}^{{\text{EM}}_{\text{sep}}}\right)+{\bar{s}}_{it}\left(x^{{\text{EM}}_{\text{sep}}}\right)+N_{i}{\bar{r}}_{i}}$$

where ${\hat{x}}_{j}^{n,m}$ is the EM image estimate at iteration n and subset m, $S_{m}$ includes all projection indices in subset m, $\phi_{itj}^{n,m}$ are adjusted sinogram update factors, and ${\bar{s}}_{it}\left(x^{{\text{EM}}_{\text{sep}}}\right)$ is scatter from the EM image alone. At the start of an EM update we set ${\hat{x}}_{j}^{0}={\hat{x}}_{j}^{\text{Input}}$, before looping over subsets at a given n we initialize ${\hat{x}}_{j}^{n,1}={\hat{x}}_{j}^{n-1}$, and after iterating over subsets we set ${\hat{x}}_{j}^{n}={\hat{x}}_{j}^{n,n_{s}+1}$. We observed a ring artifact at the edge of EM images when using (11) and setting sinogram update factors $\phi_{itj}^{n,m}={\text{Δ}}_{itj}^{n,m}$. This was likely primarily due to subtle inconsistencies between $y_{it}^{{\text{EM}}_{\text{sep}}}$ and the blank sinogram (e.g. due to count-rate variations in TOF resolution and/or pulse-pileup), which were not included in our EM update algorithm, and impacts ${\hat{x}}_{j}^{n,m}$ voxels where counts from the TX source were much greater than that from the patient. To correct for this artifact, we adjust sinogram update factors with (12).

Another difference with our algorithm and conventional OP-OSEM is that $a_{i}$ are not included in the sensitivity image; denominator in (11). The reason for this is to improve computational efficiency, as we avoid recomputing the sensitivity image, with a costly back-projection operation, after μ-map updates. The formulation in (11)–(13) and conventional OP-OSEM will produce the same result, when we set $\phi_{itj}^{n,m}={\text{Δ}}_{itj}^{n,m}$ in (11). Further, we note that the result of (11), using $\begin{array}{c} y_{it}^{{\text{EM}}_{\text{sep}}} \end{array}$, will be the same as an OSEM optimization of (4), inputting $y_{it}$, when the support of the true projection data from the subject is not clipped by the masking operation.

### 4) Transmission Reconstruction:

To reconstruct μ-map estimates we employed a penalized-likelihood algorithm. The optimal attenuation image voxels $\left(\hat{\mu }\right)$ or update factors were determined by maximizing (4) with an ordered subset implementation of the separable paraboloidal surrogates (SPS) algorithm developed by Erdogan and Fessler [22]. A nonpenalized SPS μ-map update, for ^176^Lu-aided MLAA, was first developed by Cheng et al. [21]. The μ-map reconstruction update at each subset is given by

(14) $$\begin{array}{l} {\hat{\mu }}_{j}^{n,m+1}\\ ={\hat{\mu }}_{j}^{n,m}\\ +\frac{n_{S}\sum_{i\in S_{m}}A_{ij}\left[{\dot{h}}_{i}^{\text{sum}}+\alpha {\dot{h}}_{i}^{{\text{TX}}_{\text{sep}}}\right]-\beta \dot{R}\left({\hat{\mu }}_{j}^{n,m}\right)}{n_{S}\sum_{i\in S_{m}}A_{ij}\gamma_{i}\left[c_{i}^{\text{sum}}+\alpha c_{i}^{{\text{TX}}_{\text{sep}}}\right]+2\beta R_{\psi }\left({\hat{\mu }}_{j}^{n,m}\right)} \end{array}$$

where $\dot{R}\left({\hat{\mu }}_{j}^{k}\right)$ is the derivative of the roughness penalty function, $R_{\psi }\left({\hat{\mu }}_{j}^{k}\right)$ is a separable surrogate for the penalty function, $\gamma_{i}=\sum_{j}A_{ij}$, and ${\dot{h}}_{i}$ and $c_{i}$ are functions that both input a forward projection of the current μ-map estimate $\left(l_{i}^{n,m}=\sum_{j}A_{ij}{\hat{\mu }}_{j}^{n,m}\right)$. At the start of a μ-map update we set ${\hat{\mu }}_{j}^{0}={\hat{\mu }}_{j}^{\text{Input}}$, before looping over subsets at a given n we initialize ${\hat{\mu }}_{j}^{n,1}={\hat{\mu }}_{j}^{n-1}$, and after iterating over subsets we set ${\hat{\mu }}_{j}^{n}={\hat{\mu }}_{j}^{n,n_{s}+1}$. We note that even for α=0, there is still contribution of counts originating from the TX source. Consistent with prior MLAA methods [18], [23], the transmission implementation in (14) operates on projection data summed over the TOF dimension. This non-TOF algorithm is a good approximation for a TOF scheme [24], while also substantially reducing the number of computations needed for each update. These functions are defined further for ${\dot{h}}_{i}$

(15) $$\begin{array}{l} {\dot{h}}_{i}^{\text{sum}}=\dot{h}\left(l_{i}^{n,m},y_{i}^{\text{sum}},{\bar{y}}_{i}^{\text{sum}},{\hat{b}}_{i}^{\text{sum}}\right),\\ {\dot{h}}_{i}^{{\text{TX}}_{\text{sep}}}=\dot{h}\left(l_{i}^{n,m},y_{i}^{{\text{TX}}_{\text{sep}}},{\bar{y}}_{i}^{TX_{\text{sep}}},b_{i}^{TX_{\text{sep}}}\right) \end{array}$$

(16) $$\dot{h}\left(l_{i}^{n,m},y_{i},{\bar{y}}_{i},{\hat{b}}_{i}\right)=\left(1-\frac{y_{i}}{{\bar{y}}_{i}}\right){\hat{b}}_{i}\exp\left(-l_{i}^{n,m}\right)$$

where ${\hat{b}}_{i}^{\text{sum}}=\sum_{t}b_{it}+\frac{\sum_{j}A_{ij}{\hat{x}}_{j}^{n}}{N_{j}}$, using the current EM image reconstruction, and $y_{i}^{\text{sum}}=\sum_{t}y_{it}$ and ${\bar{y}}_{i}^{\text{sum}}=\sum_{t}{\bar{y}}_{it}$ are measured and expected prompts summed over all TOF bins.

The $c_{i}$ functions are defined as in (17) and (18), where ${\bar{w}}_{i}$ is the sum of the expected randoms and scatter. These combined noise terms are given by

(17) $$c_{i}^{\text{sum}}=c\left(l_{i}^{n,m},y_{i}^{\text{sum}},{\bar{y}}_{i}^{\text{sum}},{\hat{b}}_{i}^{\text{sum}},{\bar{w}}_{i}^{\text{sum}}\right),c_{i}^{{\text{TX}}_{\text{sep}}}=c\left(l_{i}^{n,m},y_{i}^{{\text{TX}}_{\text{sep}}},{\bar{y}}_{i}^{{\text{TX}}_{\text{sep}}},b_{i}^{{\text{TX}}_{\text{sep}}},{\bar{w}}_{i}^{{\text{TX}}_{\text{sep}}}\right)$$

(18) $$c\left(l_{i},y_{i},{\bar{y}}_{i},{\hat{b}}_{i},{\bar{w}}_{i}\right)=\{\begin{array}{ll} \frac{2}{{\left(l_{i}\right)}^{2}}\left\{{\hat{b}}_{i}\left[1-\exp\left(l_{i}\right)\right]-y_{i}\log\left[\frac{{\hat{b}}_{i}+{\bar{w}}_{i}}{{\bar{y}}_{i}}\right]+l_{i}\left[\frac{y_{i}}{{\bar{y}}_{i}}-1\right]{\hat{b}}_{i}\exp\left(l_{i}\right)\right\}, & \text{if}\;l_{i}>0\\ {\hat{b}}_{i}\left(1-\frac{{\bar{w}}_{i}y_{i}}{{\left({\hat{b}}_{i}+{\bar{w}}_{i}\right)}^{2}}\right), & \text{otherwise} \end{array}$$

(19) $${\bar{w}}_{i}^{\text{sum}}=\frac{\left({\bar{s}}_{i}\left(b^{{\text{TX}}_{\text{sum}}}\right)+\sum_{t}{\bar{s}}_{it}(\hat{x})\right)}{N_{i}}+n_{t}{\bar{r}}_{i}$$

(20) $${\bar{w}}_{i}^{{\text{TX}}_{\text{sep}}}=\frac{{\bar{s}}_{i}\left(b^{{\text{TX}}_{\text{sep}}}\right)}{N_{i}}+{\bar{r}}_{i}^{{\text{TX}}_{\text{sep}}}$$

where ${\bar{s}}_{i}\left(b^{{\text{TX}}_{\text{sum}}}\right)$ is the scatter originating from the TX source summed over all TOF bins, $n_{t}$ is the number of TOF bins, and the terms in (20) are defined after (3). The update equations utilize optimal curvature, as represented by the $c_{i}$ term in (18) [22], to provide fast convergence for μ-map updates and the sTX-MLAA as a whole.

The roughness penalty function is defined as

(21) $$R(\mu )=\sum_{j=1}^{n_{j}}\sum_{k\in {\text{𝒩}}_{j}}\kappa_{jk}V\left(\mu_{j}-\mu_{k}\right)$$

where $n_{j}$ is the number of voxels in the image, ${\text{𝒩}}_{j}$ is the 26-voxel 3D neighborhood around voxel j, $\kappa_{jk}$ is the inverse Euclidean distance between the voxel coordinates j and k, and $V\left(\mu_{j}-\mu_{k}\right)$ is the edge preserving Huber function.

The Huber equation is given by

(22) $$\begin{array}{l} V\left(\mu_{j}-\mu_{k}\right)\\ =\{\begin{array}{ll} {\left(\mu_{j}-\mu_{k}\right)}^{2}/2/\delta , & \text{if}\left|\mu_{j}-\mu_{k}\right|\leq \delta \\ \left|\mu_{j}-\mu_{k}\right|-\delta /2, & \text{otherwise} \end{array} \end{array}$$

where δ was fixed at 3.0 × 10^−2^ cm^−1^ for all experiments.

We also utilized segmented TX source data alone $\left({\text{TX}}_{\text{sep}}\right)$ to reconstruct μ-maps, by modifying (14) as follows:

(23) $${\hat{\mu }}_{j}^{n,m+1}=\mu_{j}^{n,m}\;+\frac{n_{S}\sum_{i\in S_{m}}A_{ij}{\dot{h}}_{i}^{\text{TX}}-\beta \dot{R}\left({\hat{\mu }}_{j}^{n,m}\right)}{n_{S}\sum_{i\in S_{m}}A_{ij}\gamma_{i}c_{i}^{\text{TX}n\;\text{sep}}+2\beta R_{\psi }\left({\hat{\mu }}_{j}^{n,m}\right)}$$

where the terms are defined above.

### 5) sTX-MLAA Combined Update Equation:

We combined sequential EM image and attenuation map updates, as shown in pseudo-code Algorithm 1, to reconstruct μ-map estimates and the nuisance EM image.

### 6) Data Corrections:

Scattered photons originating from the TX source and patient were estimated independently using two different algorithms. For both, the single scatter simulation (SSS) method [25] was employed to estimate unscaled scatter sinograms. Our key assumptions for scatter correction are as follows: 1) in prompts segmented around the TX source $\left(y_{i}^{{\text{TX}}_{\text{sep}}}\right)$ we assume zero patient true or scattered (i.e. ${\bar{s}}_{i}\left(x^{{\text{TX}}_{\text{sep}}}\right)=0$) counts, matching the assumption of (3), and 2) for patient tracer estimates $\left({\hat{x}}_{j}\right)$, reconstructed with (11) from prompts segmented around the patient $\left(y_{it}^{{\text{EM}}_{\text{sep}}}\right)$, we assume negligible contribution from scatter originating from the TX source (i.e. ${\bar{s}}_{it}\left(b^{{\text{EM}}_{\text{sep}}}\right)=0$). We note that we do expect true coincidences from the TX source, largely at the edge of the FOV, in the radially thresholded patient prompts $\left(y_{it}^{{\text{EM}}_{\text{sep}}}\right)$ used for patient tracer updates. The majority of these counts have not undergone attenuation, and are accounted for by including the blank projection in the EM update in (11)–(13), and thus, do not violate our second scatter assumption.

#### a) Transmission scatter:

For scatter estimation of events originating from the TX source we performed the following steps: 1) reconstruct the blank sinogram with a non-TOF implementation of OP-OSEM, 2) reconstruct an initial μ-map using the radially thresholded data $\left(y_{i}^{{\text{TX}}_{\text{sep}}}\right)$ and the algorithm in (23), 3) estimate absolute (unscaled) scatter sinograms with a fully 3D implementation of the SSS method, using the reconstructed blank sinogram and initial μ-map as inputs, and 4) scale scatter estimates by tail fitting on the blank subtracted sinogram $\left(s_{i}^{{\text{TX}}_{\text{sep}}}\right)$. This blank subtracted sinogram is expected to contain only scattered events for LORs intersecting the TX source but not the attenuating material, and is given by

(24) $$s_{i}^{{\text{TX}}_{\text{sep}}}=N_{i}\left(y_{i}^{{\text{TX}}_{\text{sep}}}-b_{i}^{{\text{TX}}_{\text{sep}}}-{\bar{r}}_{i}^{{\text{TX}}_{\text{sep}}}\right).$$

**Algorithm 1:** sTX_MLAA ($\hat{\mu }$, $\hat{\text{x}}$, α, β, MLAAIter): Supplemental Transmission-Aided MLAA

1:	**Input:** $\hat{\mu }$, $\hat{x}$, α, β, and MLAAIter
2:	OSEMIter = 1, TXIter = 1
3:	**Initialize** ${\hat{\mu }}_{j}^{0}=\hat{\mu }$ and ${\hat{x}}_{j}^{0}=\hat{x}$
4:	**for** n=1 to MLAAIter **do**
5:	$a_{i}=\exp\left(-\sum_{j}A_{ij}\mu_{j}^{n-1}\right)$
6:	**Compute** $x_{j}^{n}$ with OSEM as in (11), by inputting $x_{j}^{n-1}$, $a_{i}$, and OSEMIter iterations
7:	${\hat{b}}_{i}^{\text{sum}}=b_{i}^{\text{sum}}+\frac{\sum_{j}A_{ij}{\hat{x}}_{j}^{n}}{N_{i}}$
8:	**Compute** ${\hat{\mu }}_{j}^{n}$ with TX update in (14), by inputting $\mu_{j}^{n-1}$, α, β, and TXIter iterations
9:	**end for**
10:	**return** final $\hat{\mu }$ and $\hat{x}$

To define the support of $s_{i}^{{\text{TX}}_{\text{sep}}}$ we performed a logical AND between the “triple-point” mask $\left(m_{i}^{\text{Triple}}\right)$, in (7), and a mask of “tail-regions” neighboring the attenuating material $\left(m_{i}^{\text{Tail}}\right)$; defined by the current estimate of attenuation-correction factors ($\exp\left(\sum_{j}A_{ij}{\hat{\mu }}_{j}^{n}\right)$). To compute the scale factors, linear least squares fitting was performed between the raw SSS-estimated scatter distributions and the difference sinogram in the masked tail regions across all sinogram slices (621 total scale factors).

In sum, to estimate the scatter in radially thresholded transmission sinograms, ${\bar{s}}_{i}\left(b^{{\text{TX}}_{\text{sep}}}\right)$ in (3), we utilized the above scaling approach directly. To estimate the TX scatter summed over all the TOF bins, ${\bar{s}}_{i}\left(b^{{\text{TX}}_{\text{sum}}}\right)$ in (19), we scaled ${\bar{s}}_{i}\left(b^{{\text{TX}}_{\text{sep}}}\right)$ as follows:

(25) $${\bar{s}}_{i}\left(b^{{\text{TX}}_{\text{sum}}}\right)=\left(\frac{\sum_{it}m_{i}^{\text{Triple}}m_{i}^{\text{Tail}}b_{it}}{\sum_{i}m_{i}^{\text{Tail}}b_{i}^{{\text{TX}}_{\text{sep}}}}\right){\bar{s}}_{i}\left(b^{{\text{TX}}_{\text{sep}}}\right)$$

which is equivalent to scaling by the sum of trues in the original blank sinogram, added across the TOF dimension, divided by that in the radially thresholded blank sinogram.

#### b) Patient scatter, randoms, and dead-time:

To estimate scatter originating from the patient alone, ${\bar{s}}_{it}\left(x^{{\text{EM}}_{\text{sep}}}\right)$ in (13) and ${\bar{s}}_{it}(\hat{x})$ in (19), we utilized absolute scatter estimation [26]. In this approach, the current EM image reconstruction $\left({\hat{x}}_{j}^{n}\right)$ and attenuation image were fed into a 2D implementation of the SSS. Reconstruction of the EM image and estimation of scatter with SSS was globally iterated a total of four times to refine the scatter estimate. The resulting 2D scatter sinogram was then converted to 3D with inverse single slice rebinning and used directly (i.e. without additional scaling) to produce ${\bar{s}}_{it}(\hat{x})$, or ${\bar{s}}_{it}\left(x^{{\text{EM}}_{\text{sep}}}\right)=m_{it}^{{\text{EM}}_{\text{sep}}}{\bar{s}}_{it}(\hat{x})$.

Randoms were estimated with a variance reduction algorithm [27], while dead-time was corrected at the bucket level (combination of 4 contiguous detector blocks in a ring) using singles count rates, as in [28]. The full sTX-MLAA algorithm, including global iterations to correct for scatter, is shown in Algorithm 2.

### D. Phantom Study

#### 1) Data Acquisition and Reconstruction:

We increased the coincidence window (2τ) for all PET scans to maximize recovery of external source coincidences. Default 2τ is 4.06 ns and vendor-produced sinograms have 13 TOF bins of 312 ps each. This corresponds to a maximum spatial TOF difference of 30.4 cm from the center FOV. Thus, default settings reduce the count rate of coincidences from the TX source (radial offset of 38.1 cm), as noted by Panin et al. [18], largely affecting central sinogram bins. We used 2τ=7.81 ns and performed offline binning of list-mode data to produce sinograms with up to 25 TOF bins of 312 ps each. After radial thresholding (Section II–C.2), 2τ was reduced to 6.25 ns (maximum radial offset of 48.0 cm) to produce measured prompts $\left(y_{it}\right)$ in (4), and transmission $\left({\text{TX}}_{\text{sep}}\right)$ sinograms in (10).

**Algorithm 2:** Pseudo-Code for the Complete sTX-MLAA Method With Recursive Scatter Estimation

1:	**Input:** α, β, ScatterIter, and MLAAIter
2:	**for** p=1 to ScatterIter **do**
3:	**Initialize** $\hat{\mu }$ and $\hat{x}$ with uniform values
4:	**Initial** $\hat{\mu }$ reconstruction with $y_{i}^{{\text{TX}}_{\text{sep}}}$ in (23)
5:	**If** p==1
6:	**Compute** patient scatter (${\bar{s}}_{it}(\hat{x})$, ${\bar{s}}_{it}\left(x^{{\text{EM}}_{\text{sep}}}\right)$)
7:	**Compute** TX scatter (${\bar{s}}_{it}\left(b^{{\text{TX}}_{\text{sep}}}\right)$, ${\bar{s}}_{i}\left(b^{{\text{TX}}_{\text{sum}}}\right)$)
8:	**end if**
9:	**Initial** $\hat{x}$ reconstruction with $y_{i}^{{\text{EM}}_{\text{sep}}}$ in (11)
10:	$\left(\hat{\mu },\hat{x}\right)$ := sTX_MLAA $\hat{\mu }$, $\hat{x}$, α, β, MLAAIter)
11:	**Compute** patient scatter (${\bar{s}}_{it}(\hat{x})$, ${\bar{s}}_{it}\left(x^{{\text{EM}}_{\text{sep}}}\right)$)
12:	**Compute** TX scatter (${\bar{s}}_{it}\left(b^{{\text{TX}}_{\text{sep}}}\right)$, ${\bar{s}}_{i}\left(b^{{\text{TX}}_{\text{sum}}}\right)$)
13:	** end for**
14:	**return** final $\hat{\mu }$ and $\hat{x}$

All experiments were acquired at a single patient table position, mimicking the entirety or portion of a cardiac PET exam. For CT-AC, the imaging protocol included acquisition with 120 kVp and variable mA, and μ-maps were generated using the bilinear transform method implemented in the e7tools (Siemens Medical Solutions USA, Inc.).

For sTX-MLAA we utilized the following fixed parameters: 1) 3 global scatter iterations (ScatterIter on line 2 of Algorithm 2), 2) 4 subsets for EM and TX updates, 3) 20 iterations for the initial μ-map and tracer reconstructions (lines 4 and 9, respectively, in Algorithm 2), and 4) MLAAIter set to 60. Initial images were uniform cylinders, covering the full PET FOV (transverse D=69.7 cm, axial length=22.1 cm), set to the LAC of water (9.6 × 10^−2^ cm^−1^) or ones, for μ-map and EM images, respectively. Reconstructions had 400 × 400 × 109 voxels with sizes of 2.04 × 2.04 × 2.03 mm. Critically, for all experiments the scan duration of PET data input into sTX-MLAA, matched that input into the phantom PET image reconstructions where AC was applied (as described below).

To compare different AC methods, PET images were reconstructed with TOF OP-OSEM (no PSF modeling, 2 iterations, 21 subsets), followed by smoothing with a 5 mm FWHM Gaussian (200 × 200 × 109 matrix size with 4.07 × 4.07 × 2.03 mm voxels). Input sinograms were binned to 13 TOF bins, matching the vendor default. The reconstruction protocol matched that used for cardiac evaluations at our institution and the literature [29]. For consistency, only the μ-map was substituted with all other factors remaining constant (i.e. tracer reconstructions produced as part of sTX-MLAA were not evaluated). Besides AC, corrections for scatter (absolute scatter scaling), randoms, dead-time, and normalization were included. All reconstructions were implemented with the e7tools (Siemens Medical Solutions USA, Inc.), custom C-executables, and MATLAB (MathWorks).

#### 2) NEMA IEC Body Phantom Experiments:

A NEMA IEC PET body phantom (Data Spectrum) was filled with FDG and scanned in two configurations to approximate a human torso. The phantom contains a 1) fillable background and six spheres ranging in ID from 10 to 37 mm, and 2) a cylindrical “lung insert”, filled only with polystyrene beads (i.e. no activity). FDG activity concentration ratio of all spheres to background was 4:1. The phantom was filled with a total activity and activity concentration comparable to the torso of a 70 kg adult (i.e. 370 MBq FDG injection, 60 min uptake, 20% excretion). TX source blank acquisitions were 45 min.

For the first study the phantom was imaged alone. At the start of imaging, total activity and the activity concentration in the fillable background were 28.3 MBq and 2.8 kBq/ml, respectively, and total activity in the TX source was 16.5 MBq. The phantom was scanned for a total of 30 min. PET list-mode was binned into 10 and 30 min acquisitions. These scan times are consistent with the cardiac focused table position on PET/CT, and near the lower end for PET/MR. For sTX-MLAA, the penalty-strength, β in (14), was set to 2^12^.

**Fig. 2.**
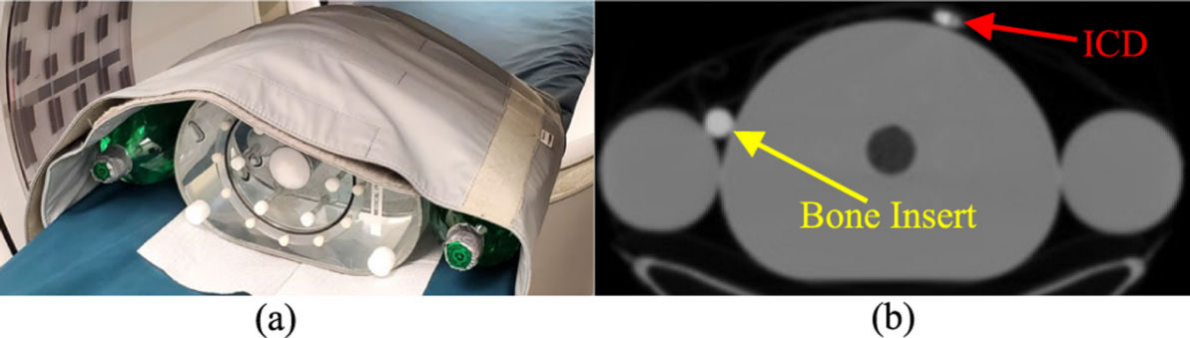
Study setup for NEMA IEC body phantom with arms, a bone insert, and ICD generator. (a) Placement of the phantom on the PET/CT table. (b) CT μ-map with display limits of 0.0 to 0.2 cm^−1^.

For the second setup, the phantom was supplemented with elements to reflect a patient with arms down and expand the range of LACs (Fig. 2). Two two-liter bottles were used for arms, while a Teflon rod insert (mimicking cortical bone) and an implantable cardioverter defibrillator (ICD) generator were positioned directly on the body phantom. At the start of imaging, total activity and the activity concentration in the torso fillable background were 20.6 MBq and 2.1 kBq/ml, respectively, and total activity in the TX source was 12.7 MBq. Acquisition time was 20 min. For sTX-MLAA, β in (14) was set to 2^14^, and reconstructed μ-maps here were post-processed by setting voxels below 1.0 × 10^−2^ cm^−1^ to zero, to minimize impact from low-intensity noisy background regions.

We quantified both absolute and relative quantification of the AC methods with an ROI analysis of EM images, conducted according to NEMA NU 2–2007 performance guidelines [30, p. 2]. Briefly, ROIs with diameters matching the inner diameters of the fillable spheres were placed on a single transverse slice, and concentric ROIs of the same diameters were placed on the background at five slices. The position of ROIs was replicated for the different reconstructions of each configuration. Sphere and background absolute uptake (in kBq/ml), sphere contrast relative to the background, the cold lung insert relative to background, and background variability were calculated.

#### 3) Anthropomorphic Torso Phantom With Cardiac Insert:

An anthropomorphic torso phantom with a cardiac insert (Data Spectrum) was prepared to approximate a patient with active cardiac sarcoidosis injected with FDG. The phantom contains fillable compartments representing organs (e.g. lungs, liver, etc.), listed in Table I, as well as a segment of myocardial inflammation. Polystyrene beads and a Teflon plastic rod are used to approximate LACs of the lungs and spine, respectively.

We filled the phantom with FDG assuming the following: 1) patient was on a specialized diet (i.e. high-fat, high-protein, low-carbohydrate) to significantly reduce non-specific myocardial uptake, 2) uptake time of 90 min and 20% tracer excretion, and 3) relative activity concentration ratios based on patient PET/CT data from our institution and prior reports [3], [31], as listed in Table I. At the start of imaging, total activity in the phantom was 26.1 MBq, background activity concentration was 1.9 kBq/ml, and TX source activity was 14.8 MBq. The TX source blank acquisition was 45 min. The phantom was scanned for 35 min, and images reconstructed for the full duration. For sTX-MLAA, β in (14) was set to 2^14^. In the same experiment, the phantom was scanned for 35 min without the TX source to assess how addition of the TX source impacts scanner dead-time. Total activity in the phantom was 31.0 MBq at the start of imaging.

#### 4) Decay Study:

To determine the minimum TX source activity needed for robust EM quantification, using AC with sTX-MLAA, we performed a decay experiment with the IEC body phantom. The TX source was filled with FDG (half-life=1.8 hrs, positron fraction=0.97) while the IEC phantom was filled with ^64^Cu (half-life=12.7 hrs, positron fraction=0.18), with an activity concentration ratio of all spheres to background of 4:1. As the half-life of ^64^Cu is >7 times that of ^18^F, this configuration enables imaging with variable TX source activity while activity in the phantom is relatively fixed. The activity concentration of ^64^Cu in the background of the IEC at the start of imaging was 13.4 kBq/ml (total activity=134.4 MBq), and was chosen to be consistent with the positron decay rate of the background for the same phantom filled with FDG (~2.8 kBq/ml) in Section II–D.2. Note, both ^64^Cu and ^18^F are pure positron emitters with positron ranges within 0.1 mm [32]. Thus, the ^64^Cu-filled phantom was expected to produce very similar count rates (i.e. trues, scatters, and randoms) as the FDG version leading to comparable EM image contrast and SNR, for all else equal [33].

The phantom was placed on the patient table (as in Section II–D.2) and scanned repeatedly for a total of 30 min, over 1.6 physical half-lives of ^18^F. A 90 min TX source blank was acquired. PET list-mode was binned into 10 min sinograms, reconstructed with sTX-MLAA, and EM image quantification assessed as in Section II–D.2.

To test our hypothesis that EM image quality for ^64^Cu and ^18^F phantom scans is matched, when the activity concentrations produce the same positron decay rates, we also performed a uniform phantom study. Duplicate uniform right cylinder phantoms (ID=21 cm) were filled with 13.6 kBq/ml of ^64^Cu and 3.1 kBq/ml of ^18^F, at the start of imaging. Phantoms were positioned on a holder at the end of the patient table. The ^64^Cu phantom was imaged for 10 min (no TX activity in place) while the ^18^F phantom was scanned repeatedly with 10 min acquisitions over a period of 70 min. Images were reconstructed with OP-OSEM using CT-AC. An 18 cm diameter VOI was placed on N=80 transverse slices, the coefficient of variation (COV) computed, and the ^18^F activity resulting in the same COV as the ^64^Cu scan computed through a regression analysis.

## III. Results

### A. NEMA IEC Body Phantom Experiments

**Fig. 3.**
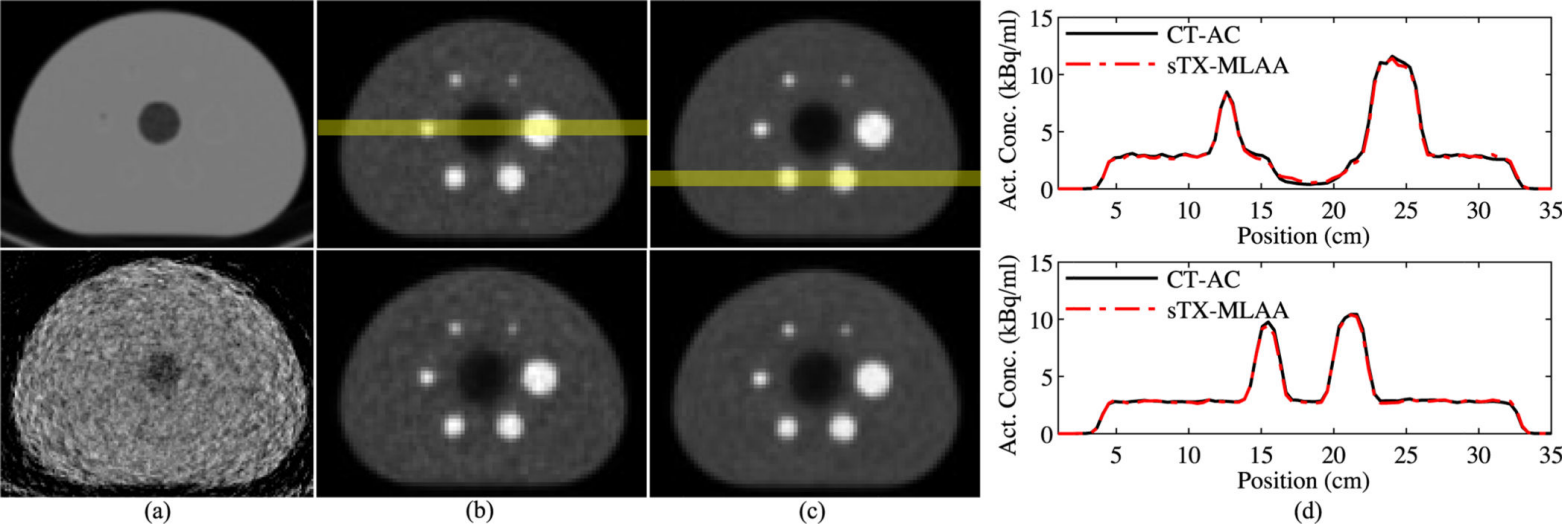
IEC phantom μ-map and PET results for two acquisition times. Transverse images of (a) μ-maps from CT (top) and sTX-MLAA for a 10 min scan (display limits=0.0–0.2 cm^−1^). (b,c) PET images generated with CT (top) and sTX-MLAA (α=10) attenuation corrections for (b) 10 min and (c) 30 min scans. (d) Line profiles from the 10 min (top) and 30 min acquisitions, with locations shown in top panels (b) and (c), respectively.

Example μ-map and PET images reconstructed with CT-based and sTX-MLAA attenuation corrections, for the IEC phantom alone, are shown in Fig. 3. Visually, PET images with AC using sTX-MLAA showed good agreement with CT-AC results. However, for sTX-MLAA PET images we did observe subtle increases in noise and/or high-frequency artifacts as well as a tendency to overestimate uptake for the cold lung insert.

**Fig. 4.**
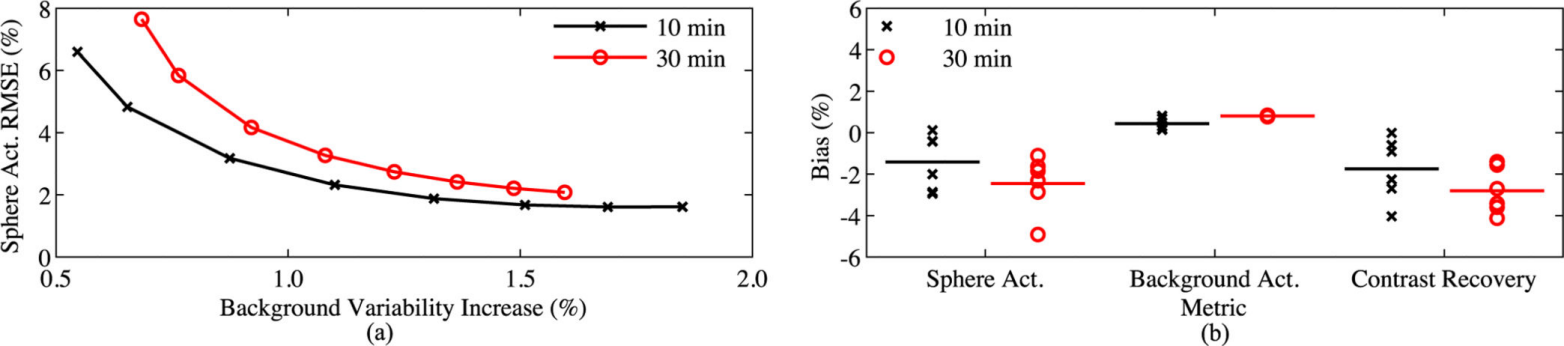
Quantification of sTX-MLAA performance for the IEC phantom and two acquisition times. (a) RMSE in measured sphere activity concentration versus increase in background variability, with respect to CT-AC PET. Each data point is for a different α in (14); range=1.0–17.5. Both the RMSE in sphere uptake and increase in background variability are across all sphere ROI sizes. (b) Bias (α=10), relative to CT-AC, in sphere activity concentration, background activity concentration, and contrast recovery. Each data point is a unique sphere ROI size and lines denote mean values.

Fig. 4 compares quantification between CT and sTX-MLAA attenuation corrected PET images at two acquisition times. As shown in Fig. 4a, the choice of α in (14), has a large impact on the root-mean-square error (RMSE) of sphere uptake bias and image noise. As α was increased from 1.0 to 17.5, sphere uptake bias decreased by ≥5% while background variability increased by ≥1%, for 10 and 30 min scans. We chose α=10 as the optimum for this study, as bias decreased by <1% compared to the lowest value at the maximum α (17.5), while variability continued increasing past this point. Fig. 4b shows bias for sTX-MLAA PET images at α=10. Sphere contrast, background contrast, and contrast recovery for sTX-MLAA were all within 4.9% of CT-AC results. Lung residual (ratio of lung to background uptake) increased from ~16.0%, with CT-AC, to 24.2% and 22.9%, with sTX-MLAA AC, for 10 and 30 min scans, respectively. Table II shows PET background variability at α=10. The RMSE increase (relative to CT-AC) was 1.3% and 1.2% for 10 min and 30 min scans, respectively.

**Fig. 5.**
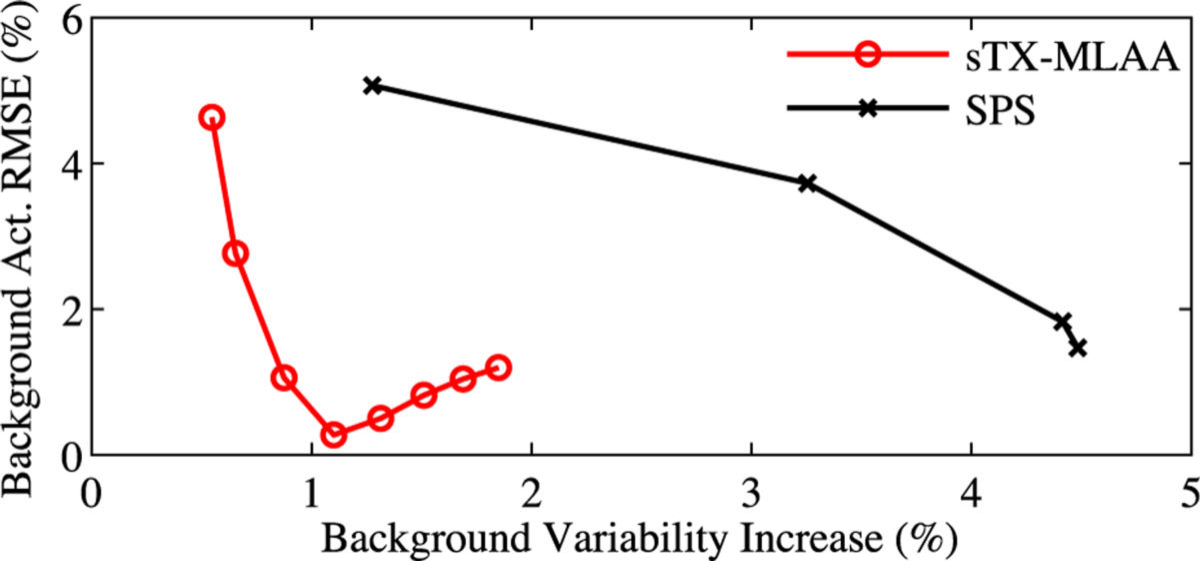
Comparison of sTX-MLAA and transmission reconstruction inputting segmented TX data only (SPS), on PET attenuation correction for the IEC phantom; 10 min scan. RMSE in measured background activity concentration versus increase in background variability, with respect to CT-AC PET, for different α in sTX-MLAA and a range of β in (23), for SPS.

Fig. 5 compares performance of sTX-MLAA against reconstructing the μ-map with the SPS algorithm in (23) using coincidences segmented from the TX source alone. We increased the iteration number of the TX reconstruction (line 4 in Algorithm 2) to 80 in the final global scatter iteration (*p* = ScatterIter) and then terminated algorithm execution (matching total TX iterations with sTX-MLAA). The penalty strength, β in (23), was varied from 2^5^ to 2^14^ in reconstructions with segmented coincidences. sTX-MLAA produced PET images with substantially lower background variability (>3.6%) compared to transmission reconstructions with segmented external coincidences, at matched background bias.

**Fig. 6.**
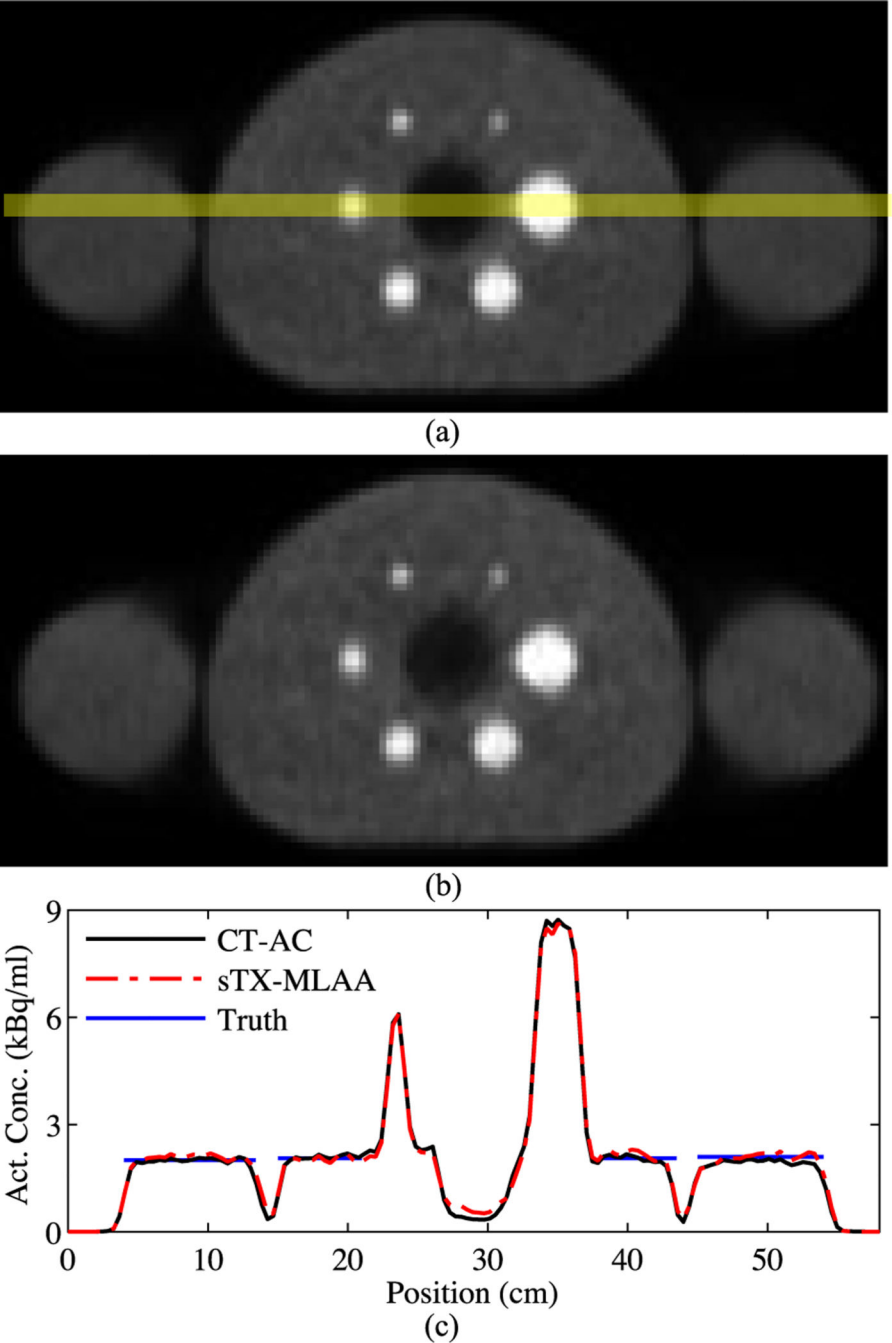
Evaluation of sTX-MLAA performance for the IEC phantom with arms, bone insert, and ICD. Transverse PET images, reconstructed with (a) CT and (b) sTX-MLAA (using α=1) attenuation correction. (c) Line profiles through the two PET reconstructions, with location shown in (a). The “Truth” profiles represent those measured with a dose calibrator. The phantom’s left arm is at position near 50 cm.

Fig. 6 qualitatively compares AC methods for the IEC body phantom supplemented with arms, a bone insert, and ICD generator. The CT μ-map, at the same slice, is shown in Fig. 2b. We found α=1, in (14), produced optimal results here. PET images reconstructed with CT-based and sTX-MLAA AC showed excellent visual agreement. However, we did again observe subtle increases in noise and/or high frequency artifacts in sTX-MLAA results, as well as positive bias in the lung insert. The phantom’s left arm slightly extended past the transverse CT FOV (D=50 cm), possibly impacting CT-AC near this position.

**Fig. 7.**
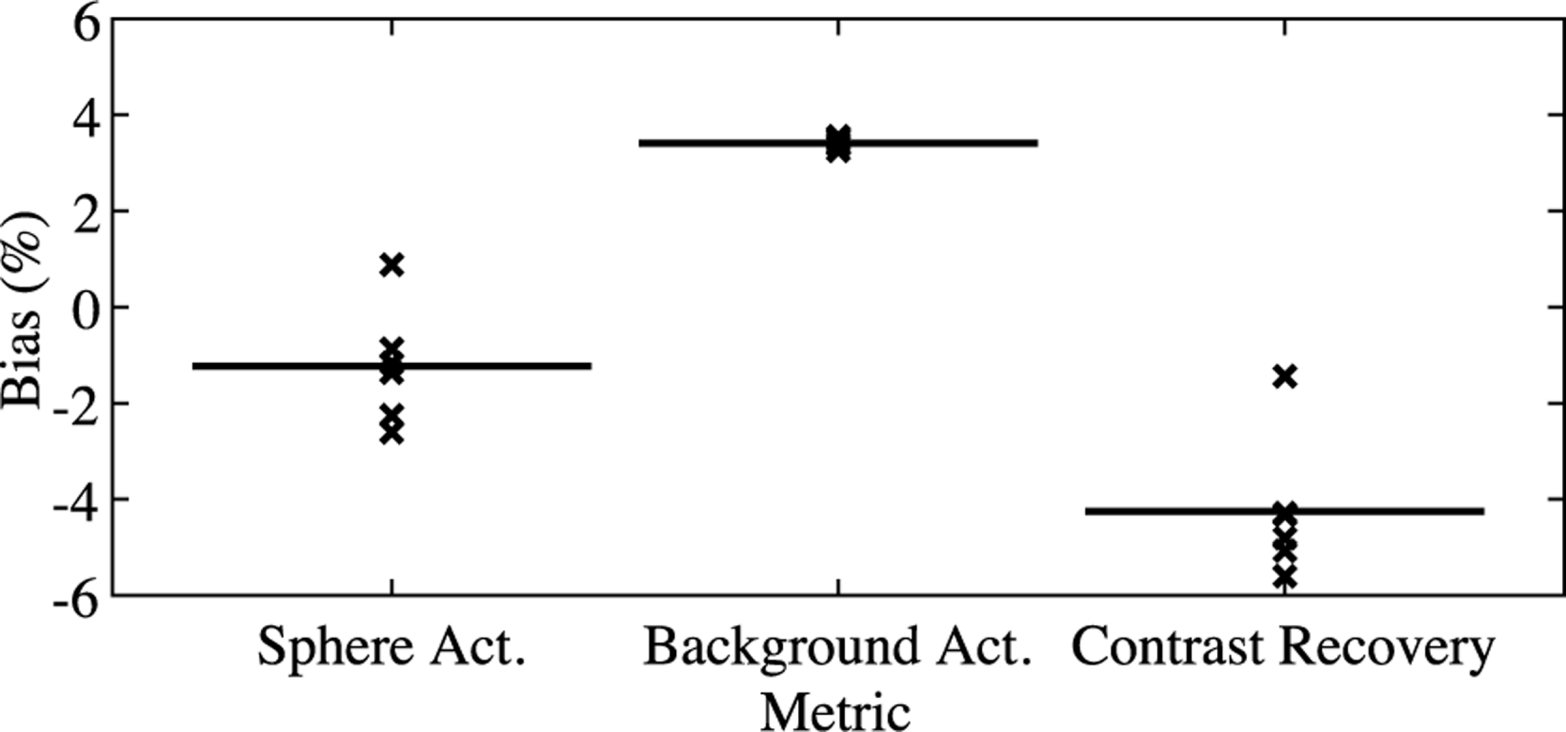
sTX-MLAA quantification for the IEC phantom with arms, bone insert, and ICD, using α=1. Bias, with respect to CT-AC results, in sphere activity concentration, background activity concentration, and contrast recovery. Each data point represents a different sphere ROI size and lines denote mean values.

Fig. 7 details the PET image ROI analysis for the IEC phantom with arms. Measured sphere and background activity concentrations for sTX-MLAA were within 3.5% (0.1 kBq/ml) of CT-AC, while bias in sphere contrast recovery was ≤ 5.6% for all spheres. Table III shows PET background variability. RMSE increase in background variability was 0.9%, and increase in the lung residual was 10.8%, for sTX-MLAA versus CT AC.

### B. Anthropomorphic Torso Phantom With Cardiac Insert

**Fig. 8.**
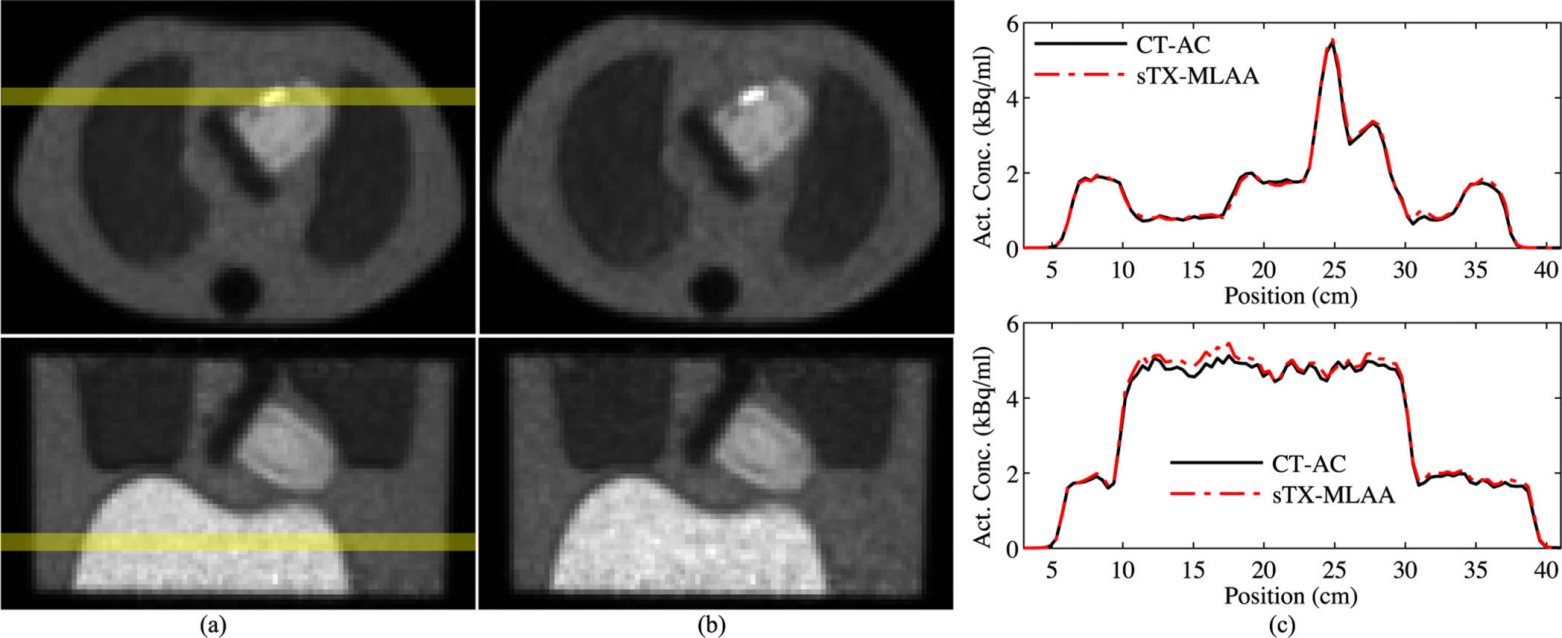
Evaluation of sTX-MLAA performance for an anthropomorphic phantom with a cardiac insert. Transverse (top) and coronal PET images, reconstructed with (a) CT and (b) sTX-MLAA (using α=10) attenuation correction. (c) Line profiles through PET images with the two AC methods for transverse (top) and coronal slices, with locations shown in top and bottom panels of (a), respectively.

Fig. 8 shows a qualitative comparison between AC methods for the phantom study modeling a patient with active cardiac sarcoidosis. We used α=10, in (14), for sTX-MLAA reconstructions. We noted high visual agreement between PET images reconstructed with CT and sTX-MLAA AC methods. As confirmed by line profile analysis (Fig. 8c), uptake in all body regions (notably the cardiac defect and lung compartments) matched closely between the two AC methods, although there was a slight overestimation in sTX-MLAA for the liver compared to CT-AC.

To quantify results further, ROIs were manually placed on transverse images of the different fillable compartments and mean uptake measured. A large spherical VOI (D=6 cm) was drawn on the liver. Table IV details these quantitative findings. Uptake in PET images corrected with sTX-MLAA was within 3.7% (0.2 kBq/ml) of CT-AC results for all organs, with the difference in liver uptake the largest recorded. Notably, the activity concentration in the cardiac defect for sTX-MLAA was only 0.9% (0.05 kBq/ml) higher than CT-AC results.

The RMSE increase in total dead-time was 2.6%, for the case with versus without the TX source in place; rising from an average of 1.6% to 4.3%. The maximum increase in dead-time was 2.9% for a block in the first detector ring, near where the transmission source helical and torus loops join (see Fig. 1).

### C. Decay Study

For the validation experiment, with uniformly filled ^64^Cu and ^18^F phantoms, we found that the ^18^F phantom scan with an activity concentration of 2.3 kBq/ml produced the same COV as the ^64^Cu phantom scan at 13.6 kBq/ml. This corresponds to a conversion factor of 0.167 ^18^F kBq/^64^Cu kBq, accounting for differences in the decay schemes and physical half-lives; 8% lower than the conversion factor (0.182 ^18^F kBq/^64^Cu kBq), accounting for positron fraction differences alone. Thus, this supports our hypothesis that EM image quality for ^64^Cu and ^18^F phantom scans is largely matched, when the activity concentrations produce the same positron decay rates.

**Fig. 9.**
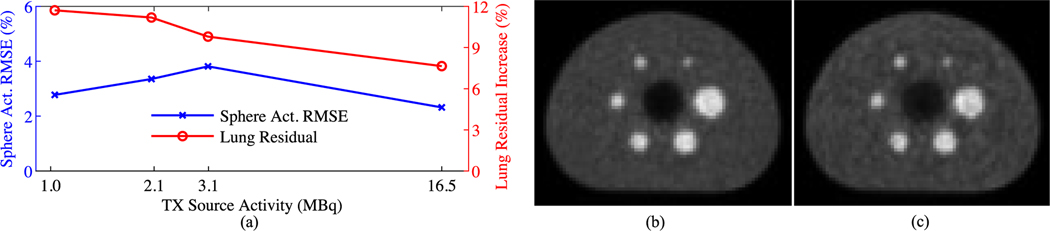
Evaluation of TX source activity on sTX-MLAA performance, for the IEC phantom. (a) RMSE in measured sphere activity (left axis) and increase in the lung residual (right axis) as a function of TX source activity (log scale), with respect to CT-AC PET (α=10.0). RMSE in sphere activity is across all sphere ROI sizes, and the data point at the TX source activity=16.5 MBq is from the FDG-filled phantom study in section III–A. Transverse PET images, reconstructed with (a) CT and (b) sTX-MLAA (TX act=3.1 MBq, α=10.0) attenuation correction.

Fig. 9 shows the impact of TX source activity on sTX-MLAA performance, compared to CT-AC. For consistency with Fig. 4, sTX-MLAA data was reconstructed with α=10 and $\beta =2^{12}$. The ^64^Cu activity concentration in the IEC phantom background changed by <15% for the TX activities considered (from 13.4 to 11.5 kBq/ml; equivalent to the count rates of ^18^F activities ranging from 2.2 to 1.9 kBq/ml). We also include results from Fig. 4, where the phantom FDG activity concentration was 2.8 kBq/ml and the TX source activity was 16.5 MBq.

As shown in Fig. 9a, the lung residual (ratio of lung to background uptake) was heavily influenced by the TX source activity, while the RMSE sphere uptake was ≤3.8% (lower limit of 2.3%) at all activities. Specifically, at a TX source activity of 16.5 MBq the increase in lung residual for AC with sTX-MLAA was 7.6%, while it was 9.8% higher at 3.1 MBq, and exceeded 11.2% for TX source activities lower than 3.1 MBq. By defining an acceptable increase in the lung residual of 10%, for this phantom study, a TX source activity of 3.1 MBq is deemed the lowest value where sTX-MLAA can produce quantitatively accurate PET images. At this lower activity limit, bias in sphere activity was ≤5.2%, background activity ≤0.7%, contrast recovery ≤6.2%, and increase in background variability 1.5%. For comparison, at a TX source activity of 16.5 MBq, bias in sphere activity was ≤2.9%, background activity ≤0.9%, contrast recovery ≤4.0%, and increase in background variability 1.4%.

## IV. Discussion

ROI results demonstrated that uptake measured in PET images corrected for attenuation with sTX-MLAA were within 5% of CT-AC results, for all phantom studies with a TX source strength ≥12.7 MBq. sTX-MLAA PET images had lower uptake bias than PET images generated with AC using coincidences largely originating from the patient alone. This was indicated by quantitative results at the lowest α values (Fig. 4a); approximating MLAA without a μ-map intensity prior (i.e. to account for LAC global scaling). Furthermore, the bias versus background variability (surrogate for noise) tradeoff for sTX-MLAA PET images was greatly improved compared to AC using coincidences segmented from the TX source alone (Fig. 5). Thus, use of patient coincidences in sTX-MLAA mitigated sparse sampling artifacts, when reconstructing μ-maps with external source events only. sTX-MLAA is a compromise approach that produces PET images with higher accuracy than MLAA (enabled by using coincidences from the TX source) and lower noise than using TX source generated μ-maps (afforded by leveraging the typically increased count rate due to tracer in the patient). Further, by utilizing transmission counts, sTX-MLAA can estimate attenuation of components not in the support of the emission image (e.g. MR coils), which also avoids the need for assumptions to correct for erroneously high air attenuation in these same regions [34].

Uptake bias in the activity-free lung insert, for experiments with the NEMA IEC body phantom, was ≥7% for sTX-MLAA versus CT-based AC PET images (Fig. 3d and Fig. 6c). A source of this error was limited tomographic sampling for the sparse TX source. The initial μ-map for sTX-MLAA (Algorithm 1) was produced by transmission reconstruction of segmented external source counts alone (line 4 in Algorithm 2), which used a starting μ-map of LACs of uniform water. Thus, voxels with low tomographic sampling in the initial TX reconstruction would overestimate LACs, which was unresolved with sTX-MLAA iterations. We note that residual activity is measured in the lung insert, even for PET images reconstructed with CT-AC [35]. Thus, lung LAC overestimation amplifies existing residual uptake. A second cause was cross-talk between emission and transmission updates, due to the residual lung uptake itself. Lung residual has been found to decrease in CT-AC results as a function of improved TOF resolution; compare the Siemens Biograph mCT [35] (TOF resolution=580 ps, lung residual=12.1%) with the Vision [36] (TOF resolution=210 ps, lung residual=3.5%), such that cross-talk in sTX-MLAA would also decrease. Although we observed uptake bias ≥7% in the IEC lung insert for sTX-MLAA, bias was only 0.7% for the lung compartment filled with an FDG activity concentration (comparable to patient imaging) in the phantom with a cardiac insert (Fig. 8c). Thus, for human FDG imaging we expect high lung uptake accuracy.

Despite promising results, there are several areas for improvement of sTX-MLAA. A key challenge is addressing noise amplification in PET images compared to CT-AC. Increased noise for sTX-MLAA is due to 1) higher randoms from addition of the TX source (not evaluated here), and 2) noise propagation to PET images from the reconstructed μ-map (increases with α). External source activity at the start of imaging for FDG phantom studies was 12.7–16.5 MBq. Our rationale for this strength was empirically determined by preliminary experiments and prior reports [13], and was expected to prioritize PET bias over noise amplification. The decay study with a ^64^Cu-filled phantom (Fig. 9) suggests that a TX activity as low as 3 MBq could be used for robust AC, but, at the cost of increased cross-talk in cold regions. Optimal activity is expected to be scanner, exam, and patient dependent, and will be explored further in future studies.

We used a penalized-likelihood transmission algorithm to improve the bias-noise tradeoff for μ-maps. A limitation is that the strength of regularization is dependent on the quality of the PET data for a fixed β in (14). As the results indicate, further noise reduction is needed. For two phantoms (IEC without arms and cardiac insert) optimal α was ~10 while it was ~1 for the IEC phantom with arms. The TX source coincidence count rate increases towards the edge of sinogram projection bins, such that a lower α may have been sufficient to correct for MLAA limitations for the IEC phantom with arms. Ideally, this parameter should be adjusted based on the PET data alone. Finally, we used absolute scatter estimation for correcting counts from the patient. The method underestimates scatter from out of FOV [26], which may bias μ-map estimation at edge planes; increasing PET bias of extracardiac disease.

We utilized a PET/CT in this study to directly compare sTX-MLAA with CT-AC performance. However, the bore diameter for whole-body PET/CT scanners (78 cm for the Biograph mCT) is generally much larger than PET/MR systems (60 cm for the Siemens Biograph mMR [37]). Thus, we would expect increased interference of true and scattered photons originating from the patient on counts segmented around the TX source $\left({\bar{y}}_{i}^{{\text{TX}}_{\text{sep}}}\right)$, with all else matched. This could increase bias in μ-map updates in (14) and (23). In general, the amount of interference depends on TOF resolution and the fraction of the transverse FOV occupied by the subject, with interference decreasing as TOF resolution improves. We did not detect interference for even the most challenging phantom study presented here, mimicking an arms down exam (Fig. 6). However, more experiments are needed to directly evaluate this assumption on PET/MR systems.

Although not compared in this report, sTX-MLAA is expected to have advantages over AC using the intrinsic radiation of ^176^Lu, particularly on the most common PET systems (i.e. with axial fields-of-view ≲30 cm). Notably, the count rate from the TX source is substantially higher than from the ^176^Lu scintillators, which may improve the bias-noise tradeoff of PET tracer quantification and reduce cross-talk. For example, with a TX source of activity of 16.5 MBq (from Fig. 4 data) we recorded a blank net trues rate of 277 kcps. In comparison, on the same PET/CT the rate for 202 and 307 keV photons from ^176^Lu is 48 kcps [15]; nearly six times lower than for the TX source. Further, sTX-MLAA fully models and corrects for scatter. A recent ^176^Lu-based AC method simply scaled the initial μ-map to compensate for scatter; contributing to PET bias often exceeding 5% [12]. Future studies are needed to directly compare the two methods.

The key benefit of sTX-MLAA is the ability to robustly estimate and correct for attenuation using data acquired simultaneously during the PET exam where AC will be applied, without use of prior results or a μ-map intensity prior (i.e. to account for LAC global scaling). This could have a substantial benefit on PET artifacts due to patient motion mismatch between the μ-map and PET acquisitions. We note that cardiac MR exams at our institution average 60 min (interquartile range: 47–79 min). Thus, if PET data were collected throughout a PET/MR study, we would expect substantial PET respiratory and non-cyclical body motion artifacts [38] when applying MR-AC generated with MR images acquired at a single timepoint. By using sTX-MLAA for AC, patient motion in the μ-map will exactly match that in tracer images; eliminating motion mismatch errors. For PET/CT, the method could decrease the radiation dose by replacing the CT-AC acquisition; important for imaging patients at higher risk from exposure to ionizing radiation [4]. Effective dose from the TX source is expected to be ~3 μ Sv for a 10 min cardiac PET/CT exam (transmission source filled with 17 MBq of ^18^F) [19], which is more than an order of magnitude lower than low-dose CT-AC protocols (0.5–1 mSv) [4].

## V. Conclusion

This paper presents a new AC strategy that uses a physically fixed and sparse transmission source that is relatively easy to fill and place. The method combines the SNR benefits of conventional MLAA, using coincidences from the patient, with the higher accuracy of transmission reconstruction with an external source. AC with sTX-MLAA produced PET images with ROI uptake within 5% of CT-AC results for phantom scans. Noise and sparse sampling artifacts were largely reduced versus AC using segmented coincidences from the external source alone. Results suggest that sTX-MLAA will enable quantitative PET during cardiac PET/MR and PET/CT of human patients.

## Figures and Tables

**TABLE I:** FDG Concentration Ratios for the Anthropomorphic Torso Phantom with Cardiac Insert

Organ	Concentration Ratio (Normalized to Background)

Background	1.0
Liver	2.7
Lungs	0.5
Myocardium	2.2
Cardiac Defect	4.9
Ventricular Blood	2.2

**TABLE II:** Background Variability in PET Images for the IEC Body Phantom with Different AC Methods

		Background Variability (%)		
		10 min.		30 min

Sphere ID	CT	sTX-MLAA	CT	sTX-MLAA

10 mm	4.1	6.2	3.0	4.8
13 mm	3.9	5.4	2.7	3.9
17 mm	3.4	4.4	2.3	3.4
22 mm	2.9	3.8	2.0	2.9
28 mm	2.5	3.4	1.8	2.8
37 mm	2.0	2.9	1.6	2.6

**TABLE III:** Background Variability in PET Images for the IEC Phantom, With Arms, for Different AC Methods

		Background Variability (%)
Sphere ID	CT	sTX-MLAA

10 mm	4.6	5.5
13 mm	4.4	5.5
17 mm	3.8	4.7
22 mm	3.6	4.5
28 mm	3.4	4.2
37 mm	3.3	4.3

**TABLE IV:** Measured Mean FDG Concentrations for the Torso Phantom With Cardiac Insert

	Activity Cone. (kBq/ml)		
Compartment	CT	sTX-MLAA	Diff. (%)^a^

Background	1.8	1.8	−0.2
Liver	4.8	5.0	3.7
Lungs	0.8	0.8	−0.7
Cardiac Defect	5.4	5.5	0.9
Ventricular Blood	3.7	3.7	−0.7

a normalized to CT-AC PET images

## References

[1] Vita T. , “Complementary value of cardiac magnetic resonance imaging and positron emission tomography/computed tomography in the assessment of cardiac sarcoidosis,” Circulat., Cardiovascular Imag., vol. 11, no. 1, Jan. 2018, Art. no. e007030. 10.1161/CIRCIMAGING.117.007030 PMC6381829 29335272

[2] Lebasnier A. , “Diagnostic value of quantitative assessment of cardiac ^18^F-fluoro-2-deoxyglucose uptake in suspected cardiac sarcoidosis,” Ann. Nucl. Med, vol. 32, no. 5, pp. 319–327, Jun. 2018. 29560563 10.1007/s12149-018-1250-3

[3] Ahmadian A. , “Quantitative interpretation of FDG PET/CT with myocardial perfusion imaging increases diagnostic information in the evaluation of cardiac sarcoidosis,” J. Nucl. Cardiol, vol. 21, no. 5, pp. 925–939, Oct. 2014. 24879453 10.1007/s12350-014-9901-9

[4] Gimelli A. , “Strategies for radiation dose reduction in nuclear cardiology and cardiac computed tomography imaging: A report from the European Association of Cardiovascular Imaging (EACVI), the Cardiovascular Committee of European Association of Nuclear Medicine (EANM), and the European Society of Cardiovascular Radiology (ESCR),” Eur. Heart J, vol. 39, no. 4, pp. 286–296, Jan. 2018. 29059384 10.1093/eurheartj/ehx582

[5] Lassen ML , “Assessment of attenuation correction for myocardial PET imaging using combined PET/MRI,” J. Nucl. Cardiol, vol. 26, no. 4, pp. 1107–1118, Aug. 2019. 29168158 10.1007/s12350-017-1118-2 PMC6660490

[6] Arabi H. and Zaidi H, “MRI-guided attenuation correction in torso PET/MRI: Assessment of segmentation-, atlas-, and deep learning-based approaches in the presence of outliers,” Magn. Reson. Med, vol. 87, no. 2, pp. 686–701, Feb. 2022. 34480771 10.1002/mrm.29003 PMC9292636

[7] Catana C, “Attenuation correction for human PET/MRI studies,” Phys. Med. Biol, vol. 65, no. 23, Dec. 2020, Art. no. 23TR02. 10.1088/1361-6560/abb0f8 PMC8590198 33263313

[8] Schaefferkoetter J. , “Deep learning for whole-body medical image generation,” Eur. J. Nucl. Med. Mol. Imag., vol. 48, no. 12, pp. 3817–3826, Nov. 2021. 10.1007/s00259-021-05413-0 34021779

[9] Ahn S. , “Joint estimation of activity and attenuation for PET using pragmatic MR-based prior: Application to clinical TOF PET/MR whole-body data for FDG and non-FDG tracers,” Phys. Med. Biol, vol. 63, no. 4, Feb. 2018, Art. no. 045006. 10.1088/1361-6560/aaa8a6 29345242

[10] Li Y, Matej S, and Karp JS, “Practical joint reconstruction of activity and attenuation with autonomous scaling for time-of-flight PET,” Phys. Med. Biol, vol. 65, no. 23, Dec. 2020, Art. no. 235037. 10.1088/1361-6560/ab8d75 PMC8383745 32340014

[11] Rezaei A, Deroose CM, Vahle T, Boada F, and Nuyts J, “Joint reconstruction of activity and attenuation in time-of-flight PET: A quantitative analysis,” J. Nucl. Med, vol. 59, no. 10, pp. 1630–1635, Oct. 2018. 29496982 10.2967/jnumed.117.204156 PMC6167531

[12] Teimoorisichani M, Panin V, Rothfuss H, Sari H, Rominger A, and Conti M, “A CT-less approach to quantitative PET imaging using the LSO intrinsic radiation for long-axial FOV PET scanners,” Med. Phys, vol. 49, no. 1, pp. 309–323, Jan. 2022. 34818446 10.1002/mp.15376 PMC9299938

[13] Mollet P, Keereman V, Bini J, Izquierdo-Garcia D, Fayad ZA, and Vandenberghe S, “Improvement of attenuation correction in time-of-flight PET/MR imaging with a positron-emitting source,” J. Nucl. Med, vol. 55, no. 2, pp. 329–336, Feb. 2014. 24434291 10.2967/jnumed.113.125989 PMC4010111

[14] Omidvari N. , “Lutetium background radiation in total-body PET—A simulation study on opportunities and challenges in PET attenuation correction,” Frontiers Nucl. Med, vol. 2, Aug. 2022, Art. no. 963067. 10.3389/fnume.2022.963067 PMC9513593 36172601

[15] Rothfuss H. , “LSO background radiation as a transmission source using time of flight,” Phys. Med. Biol, vol. 59, no. 18, pp. 5483–5500, Sep. 2014. 25163423 10.1088/0031-9155/59/18/5483

[16] Sari H. , “Quantitative evaluation of a deep learning-based framework to generate whole-body attenuation maps using LSO background radiation in long axial FOV PET scanners,” Eur. J. Nucl. Med. Mol. Imag., vol. 49, no. 13, pp. 4490–4502, Nov. 2022. 10.1007/s00259-022-05909-3 PMC9606046 35852557

[17] Chen X. and Liu C, “Deep-learning-based methods of attenuation correction for SPECT and PET,” J. Nucl. Cardiology, vol. 30, no. 5, pp. 1859–1878, Jun. 2022. 10.1007/s12350-022-03007-3 35680755

[18] Panin VY, Aykac M, and Casey ME, “Simultaneous reconstruction of emission activity and attenuation coefficient distribution from TOF data, acquired with external transmission source,” Phys. Med. Biol, vol. 58, no. 11, pp. 3649–3669, Jun. 2013. 23648397 10.1088/0031-9155/58/11/3649

[19] Bowen SL, Fuin N, Levine MA, and Catana C, “Transmission imaging for integrated PET-MR systems,” Phys. Med. Biol, vol. 61, no. 15, pp. 5547–5568, Aug. 2016. 27384608 10.1088/0031-9155/61/15/5547 PMC5004490

[20] Bowen SL, “Optimization of supplemental transmission source imaging for joint transmission-emission scanning on an integrated PET-MR system,” in Proc. IEEE Nucl. Sci. Symp. Conf., 2016.

[21] Cheng L, Ma T, Zhang X, Peng Q, Liu Y, and Qi J, “Maximum likelihood activity and attenuation estimation using both emission and transmission data with application to utilization of Lu-176 background radiation in TOF PET,” Med. Phys, vol. 47, no. 3, pp. 1067–1082, Mar. 2020. 31880818 10.1002/mp.13989

[22] Erdogan H. and Fessler JA, “Ordered subsets algorithms for transmission tomography,” Phys. Med. Biol, vol. 44, no. 11, pp. 2835–2851, Nov. 1999. 10588288 10.1088/0031-9155/44/11/311

[23] Rezaei A. , “Simultaneous reconstruction of activity and attenuation in time-of-flight PET,” IEEE Trans. Med. Imag, vol. 31, no. 12, pp. 2224–2233, Dec. 2012. 10.1109/TMI.2012.2212719 22899574

[24] Rezaei A, Michel C, Casey ME, and Nuyts J, “Simultaneous reconstruction of the activity image and registration of the CT image in TOF-PET,” Phys. Med. Biol, vol. 61, no. 4, pp. 1852–1874, Feb. 2016. 26854817 10.1088/0031-9155/61/4/1852

[25] Watson CC, Casey ME, Michel C, and Bendriem B, “Advances in scatter correction for 3D PET/CT,” in Proc. IEEE Symp. Conf. Rec. Nucl. Sci., 2004, pp. 3008–3012.

[26] Bal H, Kiser JW, Conti M, and Bowen SL, “Comparison of maximum likelihood and conventional PET scatter scaling methods for ^18^F-FDG and ^68^Ga-DOTATATE PET/CT,” Med. Phys, vol. 48, no. 8, pp. 4218–4228, Aug. 2021. 34013586 10.1002/mp.14988

[27] Byars LG , “Variance reduction on randoms from delayed coincidence histograms for the HRRT,” in Proc. IEEE Nucl. Sci. Symp. Conf. Rec., Oct. 2005, pp. 2622–2626.

[28] Casey ME, Gadagkar H, and Newport D, “Component based method for normalization in volume PET,” in Proc. Int. Meeting Fully Three-Dimens. Image Reconstr. Radiol. Nucl. Med, 1995.

[29] Norikane T, Yamamoto Y, Maeda Y, Noma T, Dobashi H, and Nishiyama Y, “Comparative evaluation of ^18^F-FLT and ^18^F-FDG for detecting cardiac and extra-cardiac thoracic involvement in patients with newly diagnosed sarcoidosis,” EJNMMI Res, vol. 7, no. 1, p. 69, Aug. 2017. 28853043 10.1186/s13550-017-0321-0 PMC5574834

[30] NEMA Standards Publication NU-2–2007: Performance Measurements of Positron Emission Tomographs, National Electrical Manufactures Association, Rosslyn, VA, USA, 2007.

[31] Coulden RA, Sonnex EP, Abele JT, and Crean AM, “Utility of FDG PET and cardiac MRI in diagnosis and monitoring of immune-suppressive treatment in cardiac sarcoidosis,” Radiol., Cardiothoracic Imag., vol. 2, no. 4, Aug. 2020, Art. no. e190140. 10.1148/ryct.2020190140 PMC7977729 33778595

[32] Conti M. and Eriksson L, “Physics of pure and non-pure positron emitters for PET: A review and a discussion,” EJNMMI Phys, vol. 3, no. 1, p. 8, Dec. 2016. 27271304 10.1186/s40658-016-0144-5 PMC4894854

[33] Braune A. , “Comparison of image quality and spatial resolution between ^18^F, ^68^Ga, and ^64^Cu phantom measurements using a digital Biograph Vision PET/CT,” EJNMMI Phys, vol. 9, no. 1, p. 58, Sep. 2022. 36064989 10.1186/s40658-022-00487-7 PMC9445107

[34] Rezaei A, Schramm G, Willekens SMA, Delso G, Van Laere K, and Nuyts J, “A quantitative evaluation of joint activity and attenuation reconstruction in TOF PET/MR brain imaging,” J. Nucl. Med, vol. 60, no. 11, pp. 1649–1655, Nov. 2019. 30979823 10.2967/jnumed.118.220871 PMC6836858

[35] Jakoby BW, Bercier Y, Conti M, Casey ME, Bendriem B, and Townsend DW, “Physical and clinical performance of the mCT time-of-flight PET/CT scanner,” Phys. Med. Biol, vol. 56, no. 8, pp. 2375–2389, Apr. 2011. 21427485 10.1088/0031-9155/56/8/004

[36] van Sluis J. , “Performance characteristics of the digital biograph vision PET/CT system,” J. Nucl. Med, vol. 60, no. 7, pp. 1031–1036, Jul. 2019. 30630944 10.2967/jnumed.118.215418

[37] Delso G. , “Performance measurements of the Siemens mMR integrated whole-body PET/MR scanner,” J. Nucl. Med, vol. 52, no. 12, pp. 1914–1922, Dec. 2011. 22080447 10.2967/jnumed.111.092726

[38] Sun T. , “Body motion detection and correction in cardiac PET: Phantom and human studies,” Med. Phys, vol. 46, no. 11, pp. 4898–4906, Nov. 2019. 31508827 10.1002/mp.13815 PMC6842053

